# Supplementary material for: Neuronal overexpression of Nrf2 reduces dystrophic neurites in 5XFAD Alzheimer’s disease model mice
Source: bioRxiv. 2026 Mar 18:2026.03.16.711596. Preprint. [Version 1] doi: 10.64898/2026.03.16.711596 (PMC13015436; doi:10.64898/2026.03.16.711596)
Supplement: Supplement 1 — Supp Figure 1: High neuronal Nrf2 overexpression increases amyloid plaque load, neuroinflammation, and neuron loss in 5XFAD mice. (A) Representative widefield microscopy images immunostained with NeuN (blue, neurons), Iba1 (green, microglia), GFAP (red, astrocytes) and MethoxyXO4 (white, dense core plaques). Size bar = 500μm. NB: Images are false colored for viewing. Imaging channels were as follows: MeXO4– 405nm, NeuN - 488nm, GFAP – 568nm, Iba1 – 647nm. Percent area covered by MeXO4 positive plaques (B) GFAP astrocytes (C) and Iba1 microglia (D) is significantly elevated in 5XFAD Nrf2 high transduced mice compared to untransduced in both cortex and hippocampus, while neuronal NeuN positive area (E) is decreased in 5XFAD Nrf2 high transduced mice in cortex and hippocampus, and in non-transgenic Nrf2 transduced mice in cortex. 2-way ANOVA with Sidak’s multiple comparisons test. * p<0.05, ** p<0.01, ***p<0.001, **** p<0.0001, one way ANOVA. Uninjected non-Tg n=6 (3F,3M), 5XFAD n=8 (4F,4M), Nrf2 high+GFP non-Tg n= 6 (3F, 3M), 5XFAD n=10 (5F, 5M). [file media-1.pdf]

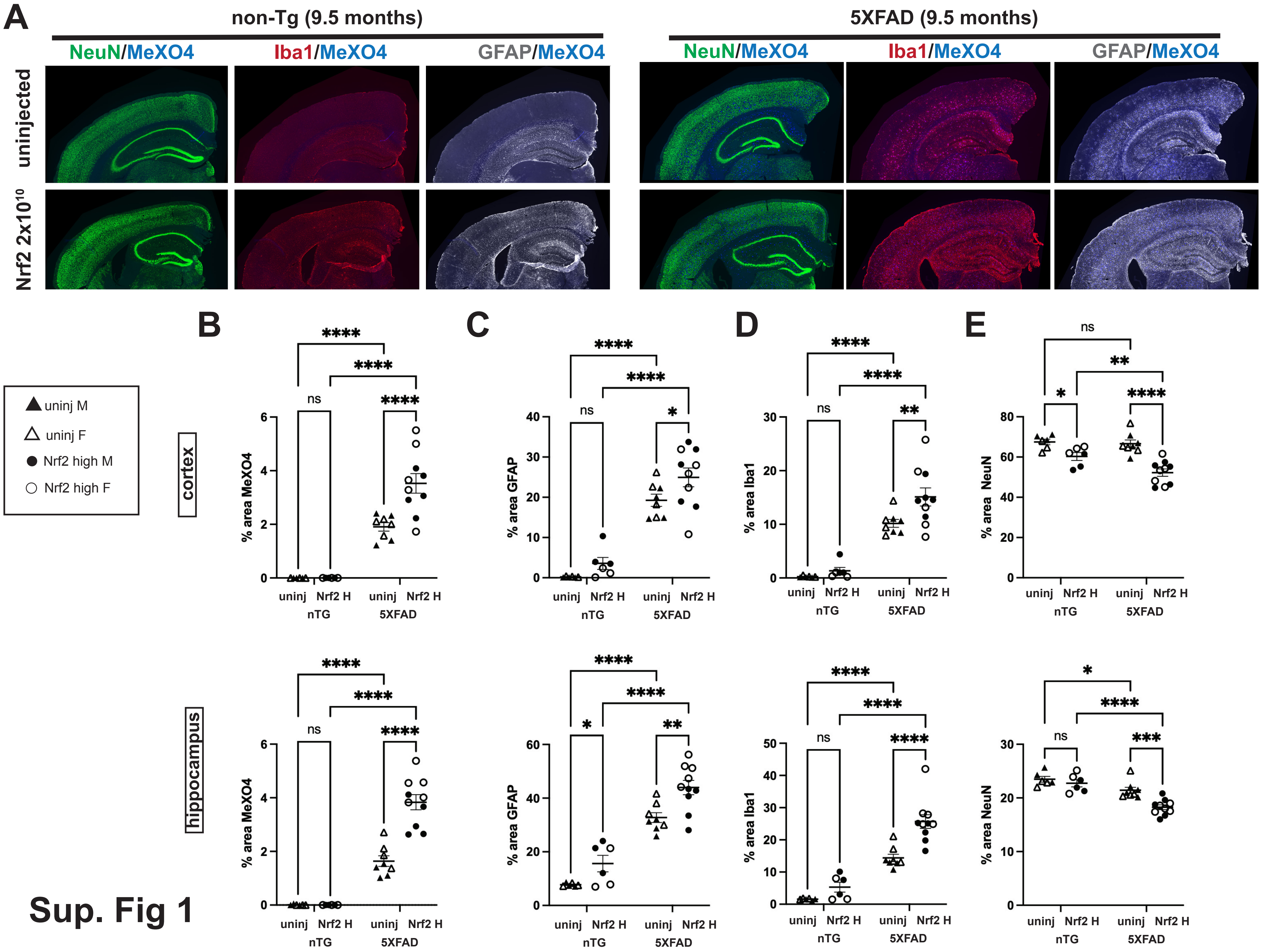
A
non-Tg (9.5 months)
5XFAD (9.5 months)
NeuN/MeXO4
Iba1/MeXO4
GFAP/MeXO4
uninjected
Nrf2 2x10^10
B
C
D
E
uninj M
uninj F
Nrf2 high M
Nrf2 high F
cortex
hippocampus
% area MeXO4
% area GFAP
% area Iba1
% area NeuN
uninj
Nrf2 H
nTG
5XFAD
ns
****
***
**
*

Sup. Fig 1

Supp Figure 1: High neuronal Nrf2 overexpression increases amyloid plaque load, neuroinflammation, and neuron loss in 5XFAD mice. (**A**) Representative widefield microscopy images immunostained with NeuN (blue, neurons), Iba1 (green, microglia), GFAP (red, astrocytes) and MethoxyXO4 (white, dense core plaques). Size bar = 500µm. NB: Images are false colored for viewing. Imaging channels were as follows: MeXO4- 405nm, NeuN - 488nm, GFAP – 568nm, Iba1 – 647nm. Percent area covered by MeXO4 positive plaques (**B**) GFAP astrocytes (**C**) and Iba1 microglia (**D**) is significantly elevated in 5XFAD Nrf2 high transduced mice compared to untransduced in both cortex and hippocampus, while neuronal NeuN positive area (**E**) is decreased in 5XFAD Nrf2 high transduced mice in cortex and hippocampus, and in non-transgenic Nrf2 transduced mice in cortex. 2-way ANOVA with Sidak's multiple comparisons test. * $p<0.05$, ** $p<0.01$, *** $p<0.001$, **** $p<0.0001$, one way ANOVA. Uninjected non-Tg *n*=6 (3F,3M), 5XFAD *n*=8 (4F,4M), Nrf2 high+GFP non-Tg *n*= 6 (3F, 3M), 5XFAD *n*=10 (5F, 5M).
